# A minimally invasive partial condylectomy and temporal bone resection for the treatment of a suspected chronic synovial sepsis of the temporomandibular joint in a 3.5-year-old paint horse gelding

**DOI:** 10.1080/01652176.2018.1535216

**Published:** 2019-02-18

**Authors:** S. K. Frietman, E. R. van Proosdij, S. Veraa, N. de Heer, F. ter Braake

**Affiliations:** a Equine Department, Equine Veterinary Clinic Emmeloord, Espelerlaan 77, Emmeloord8302DC, the Netherlands;; b Equine Department, DAP VUG, Evertsenlaan 18, Voorthuizen3781TB, the Netherlands;; c Division of Diagnostic Imaging, Utrecht University, Yalelaan 108, Utrecht3584CM, the Netherlands

**Keywords:** Equine, horse, temporomandibular joint, septic arthritis, mandibular condylectomy, arthroscopy

A 3.5-year-old Paint Horse gelding was referred to the equine hospital (Equine Veterinary Clinic Emmeloord) for a persistent painful and diffuse swelling caudo-dorsal to the right temporomandibular joint (TMJ). One-and-a-half-month prior to admission, the horse was presented to the referring veterinarian with a superficial wound to the caudal aspect of the orbit. Shortly after initial basic wound treatment, the horse developed an abscess which was drained by the owner. Since the onset of the diffuse swelling, the horse had a markedly reduced appetite. Minimal clinical improvement was observed during a three week course of procaine penicillin (Depocilline, Intervet Nederland, Boxmeer, the Netherlands) (20 mg/kg BW IM BID) and oral phenylbutazone (Butagran, Dopharma, Raamsdonksveer, the Netherlands) (2.2 mg/kg BW BID) administration. During this period, substantial weight loss was observed.

**Figure 1.**
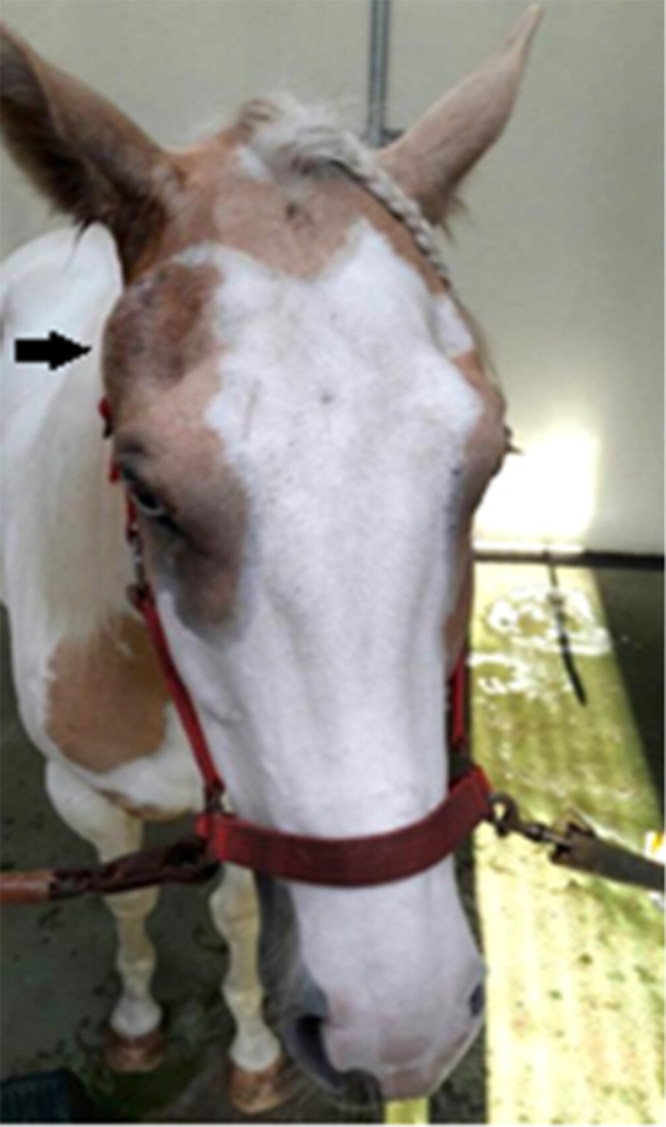
Fluctuant swelling at the level of the right TMJ (pointed by black arrow).

**Figure 2.**
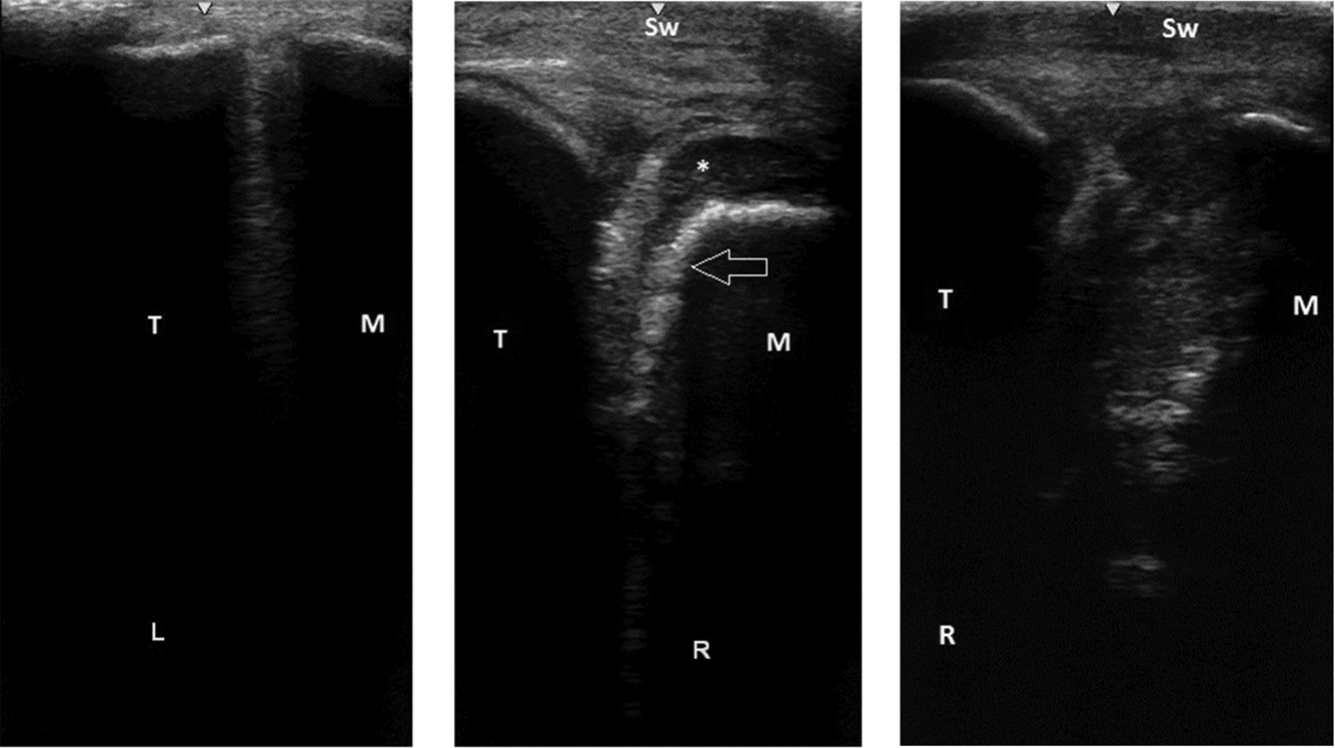
a) Ultrasonogram of the lateral aspect of the unaffected left TMJ. b) Preoperative ultrasonogram of the affected right TMJ; with clear soft tissue swelling (*Sw*), showing obvious irregular articular mandibular bone margins (Arrow) and a well-defined hypoechoic area dorsal and dorsolateral to it (*). c) Postoperative ultrasonogram, 6 weeks after arthroscopic debridement, showing a spacious right TMJ. T: Temporal bone, M: Mandible, L: Left TMJ, R: Right TMJ.

At the time of presentation to the clinic, the bodyweight of the horse was 450 kg. A complete clinical examination was performed. Physical examination of the head revealed a well-defined, fluctuant swelling at the level of the right TMJ that was painful on palpation (Figure 1). There were no evident draining tracts present. Besides some discomfort while increasing the inter-occlusal distance (opening the mouth), no oral or dental abnormalities were noted. The molar occlusal angles appeared normal and symmetric. Digital radiographic examination of the right TMJ using a 70 degree tangential projection (Townsend et al. 2009) revealed an extensive and diffuse osteolytic appearance of the lateral half of the right temporal and mandibular subchondral bone, together with moderate irregularities at the articular margins. During ultrasonographic examination (7.5 MHz linear transducer, GE Logiq e Vet, Scil Animal Care, Middelbeers, the Netherlands) the unaffected left TMJ and the affected right TMJ were compared (Figure 2a and 2b). In the affected TMJ, the articular margins were obviously irregular. A well-defined hypoechoic area dorsal and dorsolateral to the irregular discomandibular subchondral bone was visualized. A similar, but less defined area could be recognised on the ventral and ventrolateral aspect of the discotemporal subchondral bone (Figure 2b). Whether this hypoechoic area was formed by turbid synovial fluid or by necrotic and inflamed tissue remained unclear at that time. No periarticular new-bone formation was noted.

Results obtained from physical, radiographic, and ultrasonographic examinations were suggestive of a destructive septic arthritis of the TMJ. Arthroscopic debridement and lavage of the affected TMJ was proposed. Since there was a clear history of recent abscessation at the level of the right TMJ, arthrocentesis was postponed until the surgery, at which controlled conditions minimised iatrogenic inoculation of bacteria.

The gelding was sedated with detomidine hydrochloride (Detogesic, Zoetis, Capelle a/d Ijssel, the Netherlands) (10 µg/kg BW IV) and morphine hydrochloride (Morphine HCL, Centrafarm BV, Etten-leur, the Netherlands) (0.1 mg/kg BW IV) and pre-medicated with sodium benzylpenicillin (Benzylpenicilline-Natrium, Eurovet Animal Health, Bladel, the Netherlands) (40,000 IU/kg BW IV TID), gentamicin sulphate (Genta-Ject, Dopharma, Raamsdonksveer, the Netherlands) (6.6 mg/kg BW IV QID), and flunxin meglumine (Meflosyl, Zoetis, Capelle a/d Ijssel, the Netherlands) (1.1 mg/kg BW IV QID). General anaesthesia was induced using a combination of midazolam hydrochloride (Midazolam, Fresenius Kabi Nederland BV, Amersfoort, the Netherlands) (0.1 mg/kg BW IV) and ketamine hydrochloride (Anesketin, Eurovet Animal Health) (2 mg/kg BW IV).

**Figure 3.**
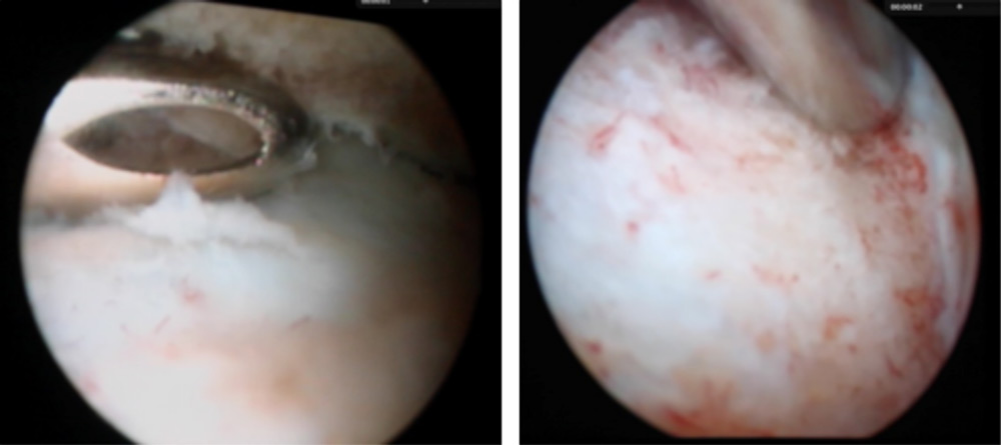
a) Intra-operative arthroscopic image, debridement of devitalized bone with a fenestrated, 3 mm, ethmoid arthroscopic rongeur (Sontec Instruments). b) debridement until healthy subchondral bone was reached.

Following induction an endotracheal tube was placed and the horse was positioned in a left lateral recumbency. Anaesthesia was maintained with isoflurane in oxygen. The surgical site was clipped and prepared for aseptic surgery. Prior to joint distension, arthrocentesis of the right TMJ was performed using a 19G hypodermic needle. Two and a half millilitres of orange coloured and turbid fluid was aspirated and submitted for synovial analysis, bacterial culture, and antimicrobial sensitivity testing. Cytology revealed a marked increase in total WBC count (127.0 G/L), % granulocytes (88%), and total protein (45 g/L). Following sterile draping, the technique described by May et al. (2001) was used for insertion of the arthroscopic cannula and obturator into the TMJ. Joint entry was confirmed by back flow of irrigation fluid. The conical obturator was replaced with a 4-mm 30° forward-viewing arthroscope (Hopkins II, Karl-Storz, Amersfoort, the Netherlands). An automatic fluid pressure control pump was used to ensure optimal visualisation. Sterile isotonic NaCl solution was used for joint irrigation. Initial arthroscopic examination demonstrated placement of the arthroscope into the caudolateral synovial pouch of the meniscomandibular compartment (MMC). Confirmation was obtained by intra-operative manipulation of the mandible, resulting in a visible excursion of the mandibular condyle relative to the disc. Intra-articular pressure was initially set at 80 mm Hg and was gradually increased to 90 mm Hg, as surgery progressed. Mild to moderate extravasation occurred. A hypodermic needle was used to ascertain the ideal location for instrument portal placement in the rostral synovial pouch of the MMC. As described by Weller et al. (2002), manoeuvrability of the arthroscope in the MMC was limited. The part of the mandibular condyle, that was arthroscopically visualised, was completely devoid of articular cartilage and partially covered with a fibrinous-like tissue. The subchondral bone consisted of friable, yellowish devitalized bone. In contrast to the pronounced cartilage loss on the mandible, the fibrocartilaginous surface of the meniscus seemed unaffected. There were signs of chronic synovitis, characterised by synovial hyperaemia, synovial hyperplasia and multiple synovial adhesions. All of the identifiable diseased tissue was resected using a size 0 and size 1 arthroscopic Bruns oval cup curettes (Sontec Instruments, Centennial, CO, USA) and a fenestrated, 3 mm, ethmoid arthroscopic rongeur (Sontec Instruments) (Figure 3a). Arthroscopic debridement was continued until hard, vital, bleeding subchondral bone was encountered (Figure 3b), followed by synovial resection using an arthroscopic shaver (FMS Tornado, Johnson & Johnson Consumer BV, Amersfoort, the Netherlands) and adhesiolysis. The arthroscope and instruments were switched several times to ensure complete debridement.

After complete debridement of the MMC, the arthroscope was inserted in the caudodorsal pouch of the meniscotemporal compartment (MTC) of the TMJ (May et al. 2001). Using the probe, placed in the rostral synovial pouch of the MMC, a clear communication between both joints could be identified. The endoscopic appearance of the articular cartilage and synovial lining of the MTC were very similar to those observed in the MMC. The articular margin of the temporal condyle was debrided in a similar fashion and all proliferative synovia were resected. A high-flow lavage of both MMC and MTC was performed at the end of the surgery. In total, 6 L of sterile NaCl solution was used. A half of a gentamycin impregnated collagen sponge (Garacol, Syntacoll GmbH, Beiern, Germany) was inserted in both joint compartments. The portals were closed with 2-0 monofilament poliglecaprone (Monocry, Ethicon Inc., Cincinnati, OH, USA) using a simple interrupted pattern. The horse’s recovery from general anaesthesia was uneventful. The overall surgery time was 1 h and 45 min. The time between induction and recovery from anaesthesia was 3 h and 10 min.

Despite the clinical findings and the macroscopic appearance of the synovial aspirate being indicative for septic arthritis, no bacterial species was cultured using a conventional agar plating technique.

The horse was hospitalized for 6 days. During this period the horse received gentamicin sulphate (Genta-ject, Dopharma, Raamsdonksveer, the Netherlands) (6.6 mg/kg BW IV QID) in combination with procaine penicillin (Depocilline, Internet Nederland BV, Boxmeer, the Netherlands) (20 mg/kg BW QID). Meloxicam (Metacam, Boehringer Ingelheim Vetmedica GmbH, Ingelheim/Rhein, Germany) (0.6 mg/kg BW PO QID) was administered for 2 weeks.

Mastication improved significantly following surgery and the horse was able to eat roughage and concentrate feed well at the time of discharge. Procaine penicillin treatment (Depocilline, Internet Nederland BV, Boxmeer, the Netherlands) (20 mg/kg BW BID) was continued for an additional 10 days at home.

Two weeks following discharge, the horse showed reduced appetite and moderate distension of the right TMJ. Synoviocentesis of the TMJ yielded approximately 1 mL of non-turbid synovial fluid with an elevated white blood cell count of 24.0 G/L and a TP of 36 g/L. Based on these findings, a thorough arthroscopic lavage was proposed. For financial reasons however, a second surgical treatment was declined by the client. The referring veterinarian administered 5 g ampicillin (Ampi-dry 5000, Dopharma, Raamsdonksveer, the Netherlands) (15 mg/kg BW) into the TMJ for three days. Besides intra-articular injection, the horse received ampicillin (Ampi-dry 5000, Dopharma, Raamsdonksveer, the Netherlands) (15 mg/kg BW TID) intravenously for 14 days. Phenylbutazone (Butagran, Dopharma, Raamsdonksveer, the Netherlands) (2.2 mg/kg BW BID) was administrated orally for 3 weeks. Clinical signs improved gradually over time and the synovial white blood cell count dropped below 1.0 G/L.

At 6 weeks, a clinical and ultrasonographic follow up was performed at the clinic. At that time, the horse did not show any abnormalities while eating roughage or concentrate feed and local swelling had reduced significantly compared to time of admission. Ultrasonography of the affected region showed a spacious TMJ without obvious joint distension (Figure 2c).

**Figure 4.**
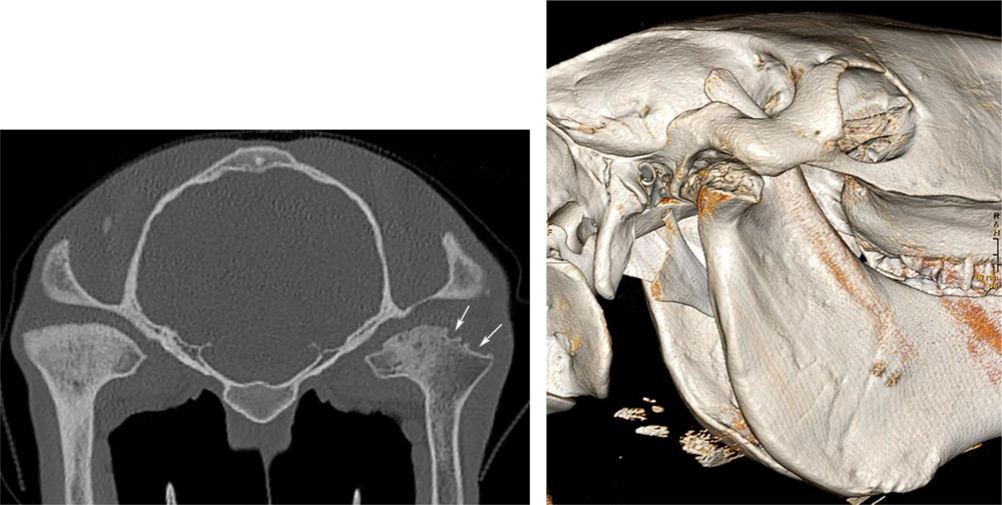
a) Transverse CT image at the level of the temporomandibular joint; right on the right side. Partial condylectomy of the mandibular condyle (white arrows) is seen. Note the disuse osteopenia of the right mandible compared to the left. b) 3-D CT image of the right temporo-mandibular joint. Note the irregular contour defect due to the partial condylectomy of the mandibular condyle.

In order to accurately document the state of the joint seven months postoperatively, the horse was referred to the Division of Diagnostic Imaging (Faculty of Veterinary Medicine, Utrecht University) for a standing sedated computed tomographic (CT) examination of the head (Somatom, Definition AS 64-slice sliding gantry, Siemens, The Hague, the Netherlands). The lateral half of the right mandibular condylar process and mandibular fossa of the articular process of the temporal bone showed an irregular but well-defined contour with substantial loss of bone and widening of the joint space. The subchondral bone of the left mandibular condylar process showed a diffuse patchy sclerosis, possibly caused by chronic overload (Figures 4a and 4b). Despite general restrictions of CT for identifying soft tissue structures, the intra-articular disc was clearly visualised.

This case report describes the successful treatment of a suspected destructive septic arthritis of the TMJ using a minimally invasive partial condylectomy technique and temporal bone resection. The horse was presented with a well-defined, painful, and fluctuant swelling of the right TMJ. Besides some signs of discomfort while manually opening the mount, no other oral or dental abnormalities were noted. Difficulties with opening the mouth, due to pain, is a common finding in horses diagnosed with TMJ-sepsis or chronic TMJ-arthropathy (Patterson et al. 1989; Weller et al. 1999; Carmalt & Wilson 2005).

Septic arthritis of the temporomandibular joint (TMJ) is relatively uncommon in horses. As in humans, infection of the TMJ in horses usually results from head trauma (Carmalt & Wilson 2005). Iatrogenic and haematogenous causes of synovial joint infection have been described in the literature, but are rarely mentioned in cases with septic arthritis of the TMJ.

Based on clinical history and physical examination, an infection of the TMJ might be suspected. Definitive diagnosis of a septic synovial infection however, is based on synovial fluid analysis and bacterial cultivation (Steel 2008). Outcome of conventional and advanced imaging techniques are supportive. Ultrasonography and radiography are readily available and frequently used in equine practice. A detailed ultrasonographic anatomy of the normal TMJ has recently been described by Weller et al. (1999) and Rodriguez et al. (2006). A specific skyline projection and a specific tangential radiographic projection, both highlighting the lateral half of the TMJ, have been reported (Ramzan et al. 2008; Townsend et al. 2009). Imaging methods such as (standing) computed tomography (CT) and magnetic resonance imaging have also become more accessible (Devine et al. 2005; Rodriguez et al. 2008; Rodriguez et al. 2010). CT examination offers excellent sensitivity and specificity for the presence of osseous abnormalities and concurrent disease of the head and more specifically the TMJ. It clearly demonstrates the extent of the joint destruction (Warmerdam et al. 1997). With the development of CT systems allowing scanning of the equine head whilst standing, the need for general anaesthesia, together with its risks, are avoided (Dakin et al. 2014; Veraa et al. 2016).

Multiple surgical methods have been reported in the literature for treatment of infectious arthritis of the TMJ. They include large open, to less invasive approaches (Carmalt & Wilson, 2005; Nagy & Simhofer, 2006; Sanders et al. 2014; Barnett et al. 2014). An open, complete, mandibular condylectomy was first described by Barber et al. (1985). Barnett et al. (2014) reported a partial mandibular condylectomy and temporal bone resection for treatment of a chronic, destructive, septic arthritis of the TMJ.

To minimise surgical trauma, new surgical techniques using minimally invasive methods have been adopted. Detailed arthroscopic approaches and (intra-articular) anatomy of the TMJ of the horse have been published (Barone 1989; May et al. 2001; Stadtbäumer & Boening 2002; Weller et al. 2002; Rodriguez et al. 2006). Successful long-term outcome after arthroscopic lavage of a septic equine TMJ has been reported (Carmalt & Wilson 2005).

Surgery in the current case was performed under general anaesthesia. Using the technique described by May et al. (2001), the arthroscope was inserted into the right TMJ. However, lack of clear landmarks complicated arthroscope placement into the caudodorsal aspect of the caudal synovial pouch of the MTC of the TMJ. After joint distension, using the most prominent part of the distended out-pouching, placement of the arthroscope into the MMC was easily accomplished. Nevertheless, in an unaffected TMJ, insertion of the arthroscope into the MMC carries the risk for iatrogenic damage of the transfacial artery and vein (May et al. 2001). No damage to the vascular structures and the adjacent salivary gland were noted while performing surgery and in the immediate postoperative period. The chronic and destructive joint disease might have facilitated insertion of the scope into the MMC by weakening of the intrinsic and extrinsic joint structures. Penetration of the parotic gland has been reported to occur in 60% of the cadaveric specimen (May et al. 2001). Ultrasonographic guided placement of the scope into the MTC and MMC reduces the risk for iatrogenic damage to the arterial, nervous and salivary structures in this area (Weller et al. 2002). Therefore, the aforementioned ultrasonographic guided technique can be considered for future arthroscopic debridement of the mandibular condyle.

After placement of the arthroscope in the caudodorsal part of the caudal pouch of the MTC and using an arthroscopic probe placed in the rostral synovial recess of the MMC, a clear communication between both joint compartments could be identified. Literature, however, remains contradictory about the presence of a communication between the MTC and MMC (May et al. 2001; Rosenstein et al. 2001; Carmalt & Wilson 2005; Rodriguez et al. 2006). In this horse, the supposed chronic septic disease along with its ongoing joint disease could have explained the communication that was arthroscopically visualized.

Carmalt and Wilson (2005) reported successful outcome using arthroscopic debridement and lavage for treatment of TMJ-sepsis in a horse. Despite some similarities, there are some important differences in surgical technique compared to Carmalt and Wilson (2005). First, the scope was also inserted into the MMC, enabling surgical debridement of the mandibular condyle. Secondly, arthroscopic debridement wasn’t limited to the intra-articular debris and proliferative synovitis, but was continued until vital subchondral bone of the articular process of the mandible and the articular process of the temporal bone was encountered.

Meticulous cleansing of the debrided area was considered crucial in preventing future impingement on the disc and therefore influencing long-term clinical outcome. Cleansing and arthroplasty techniques for the TMJ have been described in human medicine (Kirk & Kirk 2006; Kirk 2013). Carmalt et al. (2016) reported the presence of hyperdensities in the dorsorostral part of the intra-articular disc with increasing age (over 10 years), presumably caused by dystrophic mineralization associated with age-related degeneration (or “wear and tear”).

In human medicine, possible preservation of an affected meniscus is recommended as it may prevent development of postsurgical ankylosis (Li et al. 2006). Therefore, in this case, the complete disc was left in place. Surgical condylectomy, together with a complete meniscectomy has been reported to lead to pseudocondyle formation four months after surgical intervention (Nagy & Simhofer 2006). In this case report, CT examination of the head at seven months confirmed a successful partial condylectomy without signs of ankylosis or pseudocondyle formation, suggesting that preservation of the meniscus might prevent pseudocondyle formation.

Positive culture of synovial fluid samples is considered as the ‘gold-standard’ to confirm septic synovitis (Steel 2008). Furthermore, bacterial culture and sensitivity testing should drive antimicrobial selection and therefore outcome (Dumoulin et al. 2010). Although bacterial culture remained negative, history, clinical presentation, macroscopic appearance and analysis of the synovial aspirate were strongly indicative for a septic arthritis in the present case. Isolation of microorganisms from infected equine synovial fluid remains challenging (Hughes et al. 2001). Negative culture results vary between different studies and have been reported to reach up to 72% (Payne et al. 2007). Low bacterial numbers, intrinsic inhibitors in the synovial fluid and (prolonged) previous antibiotic treatment are frequently mentioned reasons causing poor culture results (Hughes et al. 2001). In the present case, the horse received a three week course of procaine penicillin prior to referral. Moreover, since there was a history of recent abcessation with persistent marked diffuse swelling, arthrocentesis was postponed until surgery, at which optimal controlled conditions minimised the risk of iatrogenic inoculation of bacteria. Regarding this case, bacterial culture might have been positive if the synovial aspirate was taken preoperative. Additionally, the use of a (automated) blood culture medium enrichment system instead of the selected conventional culture technique, could have favoured culture results. Using the latter technique, positive bacterial culture from presumed infected synovial fluid, has been reported to be 72% (Dumoulin et al. 2010).

Recurrence of clinical signs approximately 3 weeks after discharge was unexpected. Macroscopic and microscopic analysis of the synovial aspirate were not conclusive. However, a persistent low grade infection could not be excluded. A second arthroscopic lavage was proposed but was rejected by the owner, due to financial reasons. Alternatively, the horse was treated with antibiotics and anti-inflammatories for 3 weeks. The clinical signs resolved gradually within a few weeks.

Based on an anatomical study performed by Rodriguez et al. (2008), helical computed tomography appeared to be an excellent method for obtaining detailed information of the bony structures of the TMJ. Furthermore, CT examination has been shown to be extremely useful in attaining an accurate diagnosis, in preoperative planning and postoperative follow-up (Warmerdam et al. 1997). Especially in chronic, septic TMJ arthritis cases, a preoperative CT examination is strongly recommended. In particular, detailed information about the state of the medial part of the subchondral bone and possible involvement of the temporal bone is mandatory for selection of an appropriate surgical approach. In this horse, no preoperative CT scan was performed due to financial reasons at the time of referral. For scientific reasons, CT examination of the right TMJ seven months postoperative, clearly demonstrated a partial condylectomy of the mandible. The medial part of the mandibular condyle showed moderate signs of osteopenia, that presumably was caused by a prolonged disuse of the joint. However, no obvious contour abnormalities of the subchondral bone were noted.

To our knowledge, this is the first report that describes a minimally invasive partial condylectomy and temporal bone resection as a surgical option for the treatment of a suspected chronic septic arthritis of the TMJ in horses. Superior visualization and joint exploration is a considerable advantage of arthroscopic surgery. Minimally invasive approaches usually accelerate recovery time, have a reduced risk of postoperative complications and cosmetic outcome is reported to be good to excellent (McIlwraith et al. 2005; Brunsting et al. 2017). In our opinion, chronic septic arthritis of the TMJ with only lateral subchondral bone involvement of the temporal and/or mandibular bone can be successfully treated by a minimally invasive arthroscopic approach. In cases with disease of the medial subchondral bone, an open surgical approach is preferred. Since this is a single case report, further research and larger case-series with long term outcome are warranted to demonstrate that arthroscopic surgery is a viable option for the treatment of chronic septic arthritis of the TMJ with only lateral subchondral bone involvement.
